# Electronic and optical properties of chloropicrin adsorbed ZnS nanotubes: first principle analysis

**DOI:** 10.3762/bjnano.16.87

**Published:** 2025-07-25

**Authors:** Prakash Yadav, Boddepalli SanthiBhushan, Anurag Srivastava

**Affiliations:** 1 Advanced Material Research Group, Department of Engineering Sciences, ABV-Indian Institute of Information Technology and Management, Gwalior, MP-474015, India; 2 Department of Electronics and Communication Engineering, Indian Institute of Information Technology, Allahabad, UP-211015, India

**Keywords:** chemical sensor, chloropicrin (CP), density functional theory, zinc sulfide nanotubes (ZnS NTs)

## Abstract

Zinc sulfide nanotubes have garnered significant attention as potential candidates for chemical sensing applications owing to their exceptional structural, electronic, and optical properties. In this study, we employed density functional theory (DFT) to explore the sensing capabilities of a ZnS (3,3) nanotube (ZnS NT) for detecting chloropicrin (CP, CCl_3_NO_2_), a highly toxic gas. To elucidate the sensing mechanism, we systematically analyze the adsorption configurations, Mulliken charge transfer, band structure, density of states, optical absorption, and optical conductivity of the ZnS NT-CP system. Our findings reveal that the interaction between CP and ZnS NT induces notable changes in the electronic and optical properties of the nanotube, including a substantial bandgap reduction of up to ≈40% for the specific orientation A. The adsorption energy ranges from −0.389 to −0.657 eV, indicating weak physisorption. The Mulliken charge transfer varies between 0.06*e* and 0.109*e*, confirming effective but nondestructive interaction. A favorable recovery time of ≈3.533 μs at room temperature, along with a significant red shift in the absorption spectra and optical conductivity peaks, highlight the potential of ZnS NT for designing sensitive and reusable CP gas sensors.

## Introduction

Chloropicrin (CP), also known as trichloronitromethane (CCl_3_NO_2_), is a chemical warfare agent that poses significant risks of accidental, occupational, and intentional exposure [1–2]. It was first synthesized in 1848 by the Scottish chemist John Stenhouse via the reaction of picric acid with sodium hypochlorite [3]. CP is classified as a Category I toxic agent and is listed as a Schedule II substance under the Chemical Weapons Convention [4]. While its classification permits legal production, storage, and transportation for non-warfare applications, the compound remains a potential hazard due to its dual-use nature.

**Figure 1:**
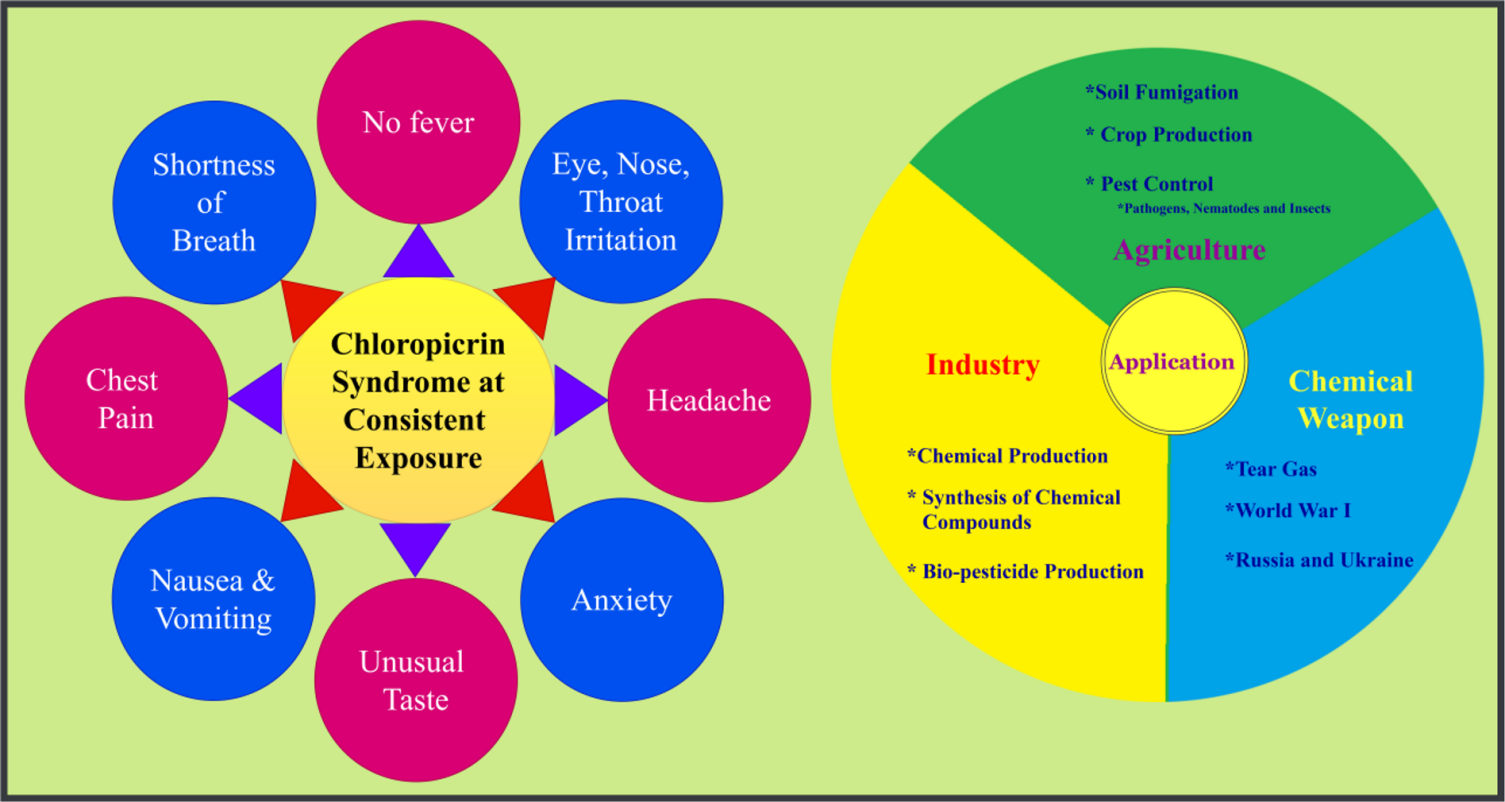
Syndrome at consistent exposure and applications of chloropicrin gas.

Recognized as both a choking agent and a pulmonary toxicant, CP exhibits potent lachrymatory and ocular irritant properties. Its degradation products, including chlorine, phosgene, nitric oxides, and ammonia, contribute to its toxicity by causing severe pulmonary and ocular damage [5]. Exposure to CP, as depicted in Figure 1, can result in respiratory injuries such as dyspnea, upper respiratory tract damage, and chest pain, even at low concentrations. Severe eye irritation is common, with the corneal epithelium being particularly vulnerable as tear fluid accumulates CP, exacerbating damage. Ocular symptoms can appear within 24 hours of exposure, with studies reporting that 99% of individuals exposed to CP experience ocular symptoms, including inflammation, corneal edema, tissue damage, and potential visual impairment [6–8].

Developing portable, sensitive, rapid-response, and reliable sensors for detecting chemical warfare agents is paramount to ensure safety in agricultural settings and protect public health from potential hazards. Nanomaterials have emerged as an exceptional class of materials, characterized by at least one dimension in the range of 1 to 100 nm. These materials exhibit remarkably high surface areas, which can be tailored through rational design. By precisely controlling their size, shape, synthesis conditions, and functionalization, nanomaterials can achieve extraordinary magnetic, electrical, optical, mechanical, sensing, anticancer, and photocatalytic properties that significantly differ from their bulk counterparts [9–14].

Zinc sulfide (ZnS) attracts considerable attention among various nanomaterials due to its unique properties. Nanostructured ZnS with different morphologies, including nanotubes, nanowires, nanoparticles, and nanosheets has been extensively investigated for applications ranging from ultraviolet light-emitting diodes and injection lasers to flat-panel displays and sensors [15–19]. ZnS, a promising transition metal chalcogenide with a wide bandgap of approximately 3.7 eV, has shown remarkable potential in gas sensing applications. Semiconductor nanomaterials such as graphene, B_12_N_12_, fullerene C_60_, carbon nanotubes, WO₃, ZnO, ZnS, ZnSe, ZnTe, SnO₂, TiO₂, MoS₂, and NiO have been widely employed in gas sensor applications due to their superior selectivity, sensitivity, and response characteristics [20–35]. ZnS NTs are well-suited for gas sensing applications owing to their unique features. These include a high surface area for enhanced gas adsorption, semiconducting properties that enable measurable conductivity changes in the presence of target gases, and their ability to form composites with other materials, such as carbon nanotubes, to optimize performance [26]. Furthermore, the flexible synthesis of ZnS NTs with controlled morphology and size allows for tailoring their sensing capabilities. External stimuli combined with machine learning can further enhance their sensitivity and selectivity for specific gases [36]. These attributes make ZnS NTs highly attractive for diverse applications, including detecting volatile organic compounds, choking and pulmonary toxicants, medical diagnostics, and developing portable and wearable gas sensing devices [18,37–45].

Previous studies have demonstrated the gas-sensing capabilities of ZnS NTs. Khan et al. [40] investigated ZnS NTs as sensors for ammonia and phosphine using density functional theory (DFT) to analyze adsorption behavior. Zhang et al. [46–47] reported that ZnS NTs exhibit superior humidity sensing performance compared to that of ZnO/ZnS nanorod arrays and ZnO nanorod arrays, with enhanced response, faster recovery, good linearity, and reliable reproducibility across a wide range of relative humidity conditions at room temperature. Giri et al. [48] demonstrated that phase-selective ZnO@ZnS heterostructures and ZnS NTs exhibited superior amperometric cholesterol sensing performance, with ZnS NTs achieving the highest sensitivity (598 mA/m^2^) and low detection limits among the configurations studied.

Despite these advances, the use of ZnS NT for detecting chloropicrin (CP), a highly toxic chemical warfare agent, remains an area with untapped potential. Addressing this gap, the present work constructs an armchair ZnS NT to investigate its adsorption configurations, charge transfer, band structure, density of states, optical absorption, and optical conductivity using a density-functional-theory-based approach. Our findings suggest that ZnS NT is a promising candidate for developing advanced sensors for detecting chemical warfare agents, with potential applications in safety, environmental monitoring, and public health protection.

## Computational Methodology

The computational analyses were conducted using the linear combination of atomic orbitals (LCAO) DFT approach, as implemented in the Synopsys-QuantumATK software [49]. Electron exchange/correlation interaction energies were calculated using the generalized gradient approximation (GGA) with the revised Perdew–Burke–Ernzerhof (RPBE) parameterization [50]. Pseudo-atomic double zeta double polarized (DZDP) basis sets were utilized to define the atomic orbitals. Brillouin zone sampling was performed using a Monkhorst–Pack grid of k-points set to 1 × 1 × 100, ensuring accurate representation of periodic boundary conditions along the axis of the nanotube. A density mesh cutoff of 150 Rydberg was applied to define the real-space grid for energy calculations.

The ZnS NT model, consisting of 36 atoms periodically arranged along the Z-direction, was structurally optimized to achieve geometric and energetic stability. Optimization was performed with stringent convergence criteria: a maximum force tolerance of 0.05 eV/Å and a stress tolerance of 0.05 eV/Å^3^. These parameters ensured the reliability and accuracy of the resulting structural configurations for subsequent electronic and transport property analyses.

To study the optical properties of the ZnS NT without and with molecular adsorption, we calculated the absorption coefficient (α) and optical conductivity σ, which can be obtained by the following formulas [21]:


[1] $$\alpha =\sqrt{2}\frac{\omega }{c}{\left(\sqrt{\varepsilon_{1}^{2}+\varepsilon_{2}^{2}}-\varepsilon_{1}\right)}^{\frac{1}{2}},$$

[2] $$\sigma =\frac{n\alpha c}{4\pi },$$

where ω is the angular frequency of light, ε_1_ and ε_2_ are real and imaginary parts of the complex permittivity, *n* is the refractive index of the materials, and *c* is the speed of light. The fundamental part of the optical conductivity is related to light absorption, while the imaginary part is associated with the dispersion.

## Results and Discussion

### Structural analysis

**Figure 2:**
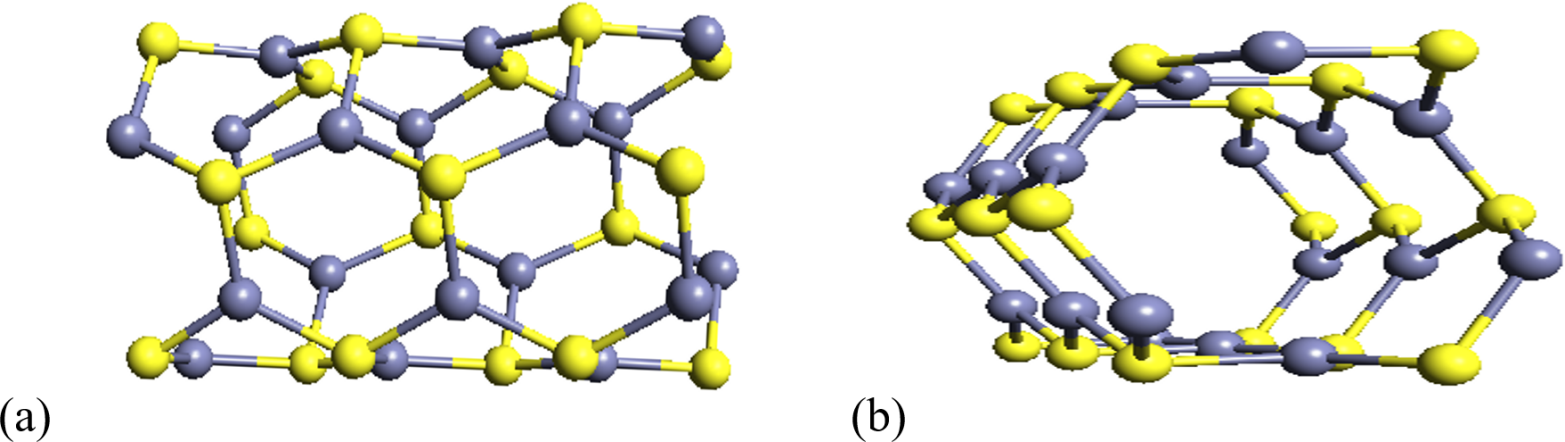
Optimized pristine ZnS NT: (a) side view, (b) front view.

The optimized geometry of the armchair ZnS NT with (3,3) chirality is illustrated in Figure 2. The structural analysis reveals an average Zn–S bond length of 2.29 Å, which is slightly shorter than the bond length of bulk ZnS (2.34 Å). This reduction in bond length can be attributed to the curvature of the NT structure, which induces a slight strain in the lattice. A vacuum slab of 20 Å thickness was introduced along the radial direction to minimize spurious interactions between adjacent NT images in the simulation. This ensures that the periodic boundary conditions do not artificially influence the electronic and structural properties of the NT.

The cohesive energy (*E*_C_) per atom of ZnS NT is calculated using the Equation 3 [51–53],


[3] $$E_{\text{C}}=\frac{1}{a+b}\left[E_{\text{ZnS NT}}-aE_{\text{Zn}}-bE_{\text{S}}\right],$$

where, *E*_ZnS NT_, *E*_Zn_ and *E*_S_ illustrate the corresponding energy of pristine ZnS NT, isolated Zn and S atoms, respectively. The number of Zn and S atoms in pristine ZnS NTs is called “a” and “b”, respectively.

Table 1 shows *E*_C_, which is the energy required to break the NT into individual atoms, the average diameter between two opposite sulfur (*d*_S_) and zinc (*d*_Zn_) atoms, and the radial buckling δ.

**Table 1:** The cohesive energy (*E*_C_), average diameter between two opposite sulfur (*d*_S_) and zinc (*d*_Zn_) atoms, and the radial buckling δ of the armchair ZnS NT.

System	Cohesive energy *E*_C_ (eV/atom)	*d*_S_ (nm)	*d*_Zn_ (nm)	δ (nm)

ZnS NT (3,3)	−3.832	0.708	0.618	0.09

The cohesive energy of the ZnS NT (ZnS NT) is calculated to be −3.832 eV/atom. This negative energy indicates that the formation of ZnS NT from its constituent elements is energetically favorable, suggesting an exothermic process. In such reactions, the energy required to break the bonds in the reactants is less than the energy released during the formation of new bonds in the product, confirming the thermodynamic stability of the NT structure. Although these NTs are derived from flat hexagonal ZnS sheets, the resulting structures exhibit slight deviations from perfect smoothness. Specifically, a small radial anion–cation buckling of approximately ±0.1 Å was observed, consistent with previous reports on reconstructed ZnS NTs [38,54–56]. This minor buckling is attributed to the inherent curvature induced during the transition from a planar sheet to a tubular form.

The presence of buckling in the NT structure is of significant interest due to its potential implications for surface properties. The buckling could form a surface dipole, enhancing the interaction of the NT with external molecules. Such structural features are particularly relevant for potential applications in gas sensing, where surface interactions play a critical role in sensitivity and selectivity.

The adsorption energy *E*_ads_ for CP with different adsorption orientations on pristine ZnS NT was calculated using Equation 4 [40].


[4] $$E_{\text{ads}}=E_{\text{(CP adsorbed ZnS NT)}}-\left(E_{\text{ZnS NT}}+E_{\text{CP}}\right),$$

where *E*_(CP adsorbed ZnS NT)_, *E*_ZnS NT_, and *E*_CP_ correspond to the energies of CP adsorbed ZnS NT, isolated ZnS NT, and isolated CP, respectively.

**Figure 3:**
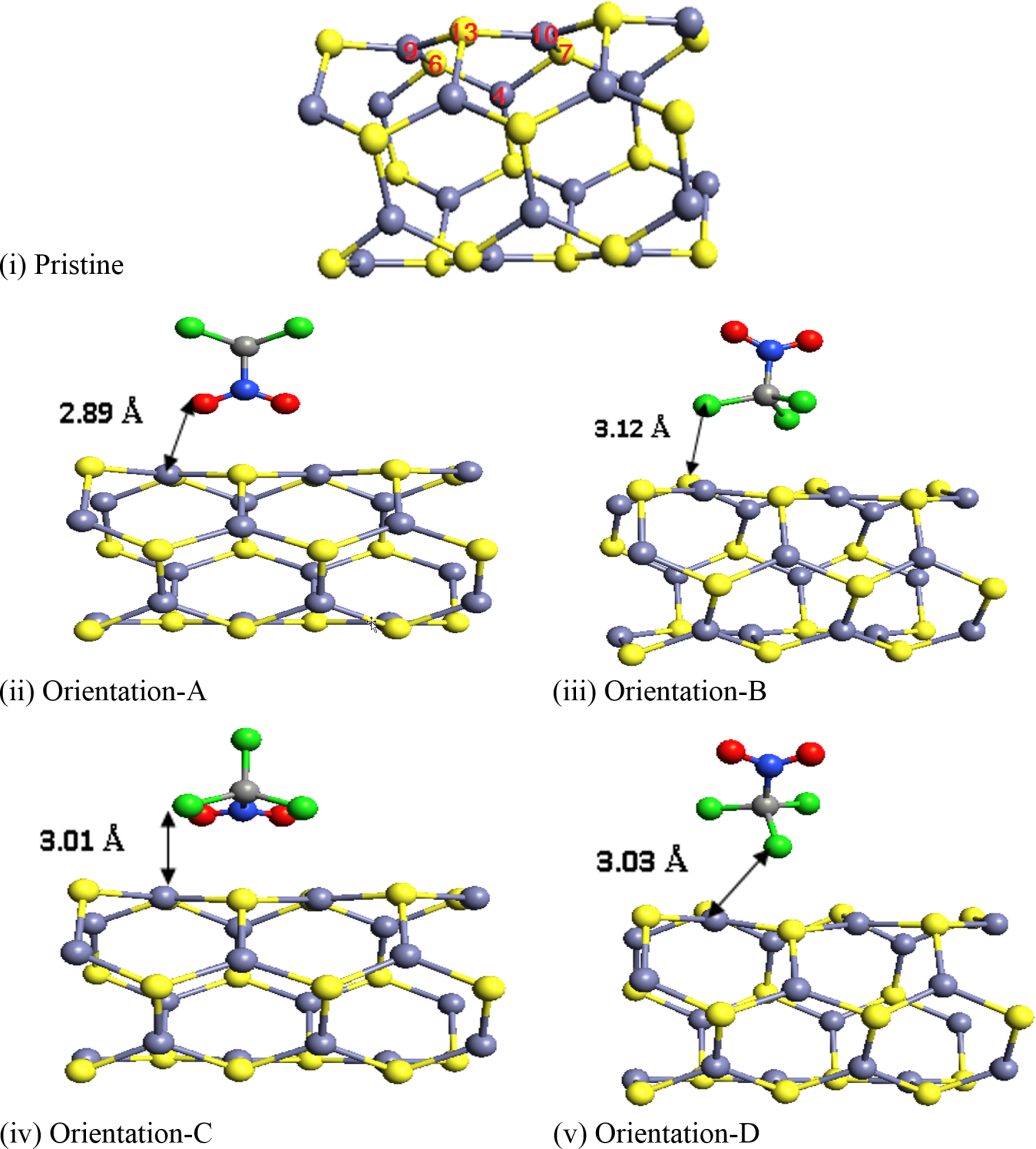
Optimized geometries of CP adsorption on ZnS NT: (i) Pristine ZnS NT, (ii) CP adsorbed ZnS NT (Orientation A), (iii) CP adsorbed ZnS NT (Orientation B), (iv) CP adsorbed ZnS NT (Orientation C), and (v) CP adsorbed ZnS NT (Orientation D). Yellow and light grey spheres represent sulfur and zinc atoms, respectively, while red, green, blue, and dark grey spheres indicate oxygen, chlorine, nitrogen, and carbon atoms of the CP molecule.

The adsorption behavior of CP on the ZnS NT surface was analyzed for four distinct molecular orientations, denoted as A, B, C, and D in Figure 3. Adsorption energies for these orientations are calculated and presented below in Table 4, with values of −0.389, −0.657, −0.593, and −0.440 eV for A, B, C, and D orientations driven by the physisorption phenomenon, respectively. Among the four orientations, B is the most favorable one with the lowest adsorption energy due to the interaction between the chlorine atoms of CP and the Zn site of ZnS NT. It is to be noted from the different orientations that the chlorine atoms of CP tend to strongly interact with the ZnS NT surface in comparison to the oxygen atoms of CP, which may be attributed to the availability of three lone pairs with each chlorine atom in comparison to the two lone pairs with each oxygen atoms. To gain deeper insight into these interactions, a Mulliken population analysis was performed, revealing the extent of electronic charge transfer from CP to ZnS NT for each molecule orientation (see below Table 4). The corresponding charge transfers are 0.073*e*, 0.095*e*, 0.109*e*, and 0.06*e* for orientations A, B, C, and D, respectively.

The electronic charge transfer led by the weak physisorption phenomenon plays a critical role in altering the electronic and optical properties of ZnS NT. A higher charge transfer improves sensor sensitivity, allowing the detection of CP at lower concentrations, whereas the weak physisorption led by the van der Waals forces may help with the reusability of sensor devices.

Table 2 highlights the changes in Zn–S bond lengths near the CP adsorption site for different orientations before and after optimization, alongside the adsorption distance between the adsorbate and adsorbent. The most significant changes in bond length were observed near the atomic positions Zn(10)–S(13)–Zn(9), which correspond to the maximum structural deformation caused by CP physisorption. These results are consistent with the adsorption energy trends.

**Table 2:** Variations in the Zn–S bond length of ZnS NT near the CP adsorption site.

S. No.	Bond length between the Zn–S atomic positions	Pristine ZnS NT	Orientation A		Orientation B		Orientation C		Orientation D	
		bond length (in Å) before optimization	bond length (in Å)	change bond length dl (in Å)	bond length (in Å)	change bond length dl (in Å)	bond length (in Å)	change bond length dl (in Å)	bond length (in Å)	change bond length dl (in Å)
			after optimization		after optimization		after optimization		after optimization	

1	Zn–S (13-9)	2.324	2.319	0.005	2.309	0.015	2.312	0.012	2.310	0.014
2	Zn–S (9-6)	2.267	2.273	−0.006	2.270	−0.003	2.256	0.011	2.266	0.001
3	Zn–S (6-4)	2.316	2.319	−0.003	2.305	0.011	2.305	0.011	2.316	0
4	Zn–S (4-7)	2.317	2.319	−0.002	2.313	0.004	2.309	0.008	2.324	−0.007
5	Zn–S (7-10)	2.265	2.281	−0.016	2.263	0.002	2.256	0.009	2.266	−0.001
6	Zn–S (10-13)	2.321	2.316	0.005	2.302	0.019	2.309	0.012	2.295	0.026
7	adsorption distance of CP molecule		2.89 Å		3.01 Å		3.12 Å		3.03 Å	

### Band structures and density of states analysis

**Figure 4:**
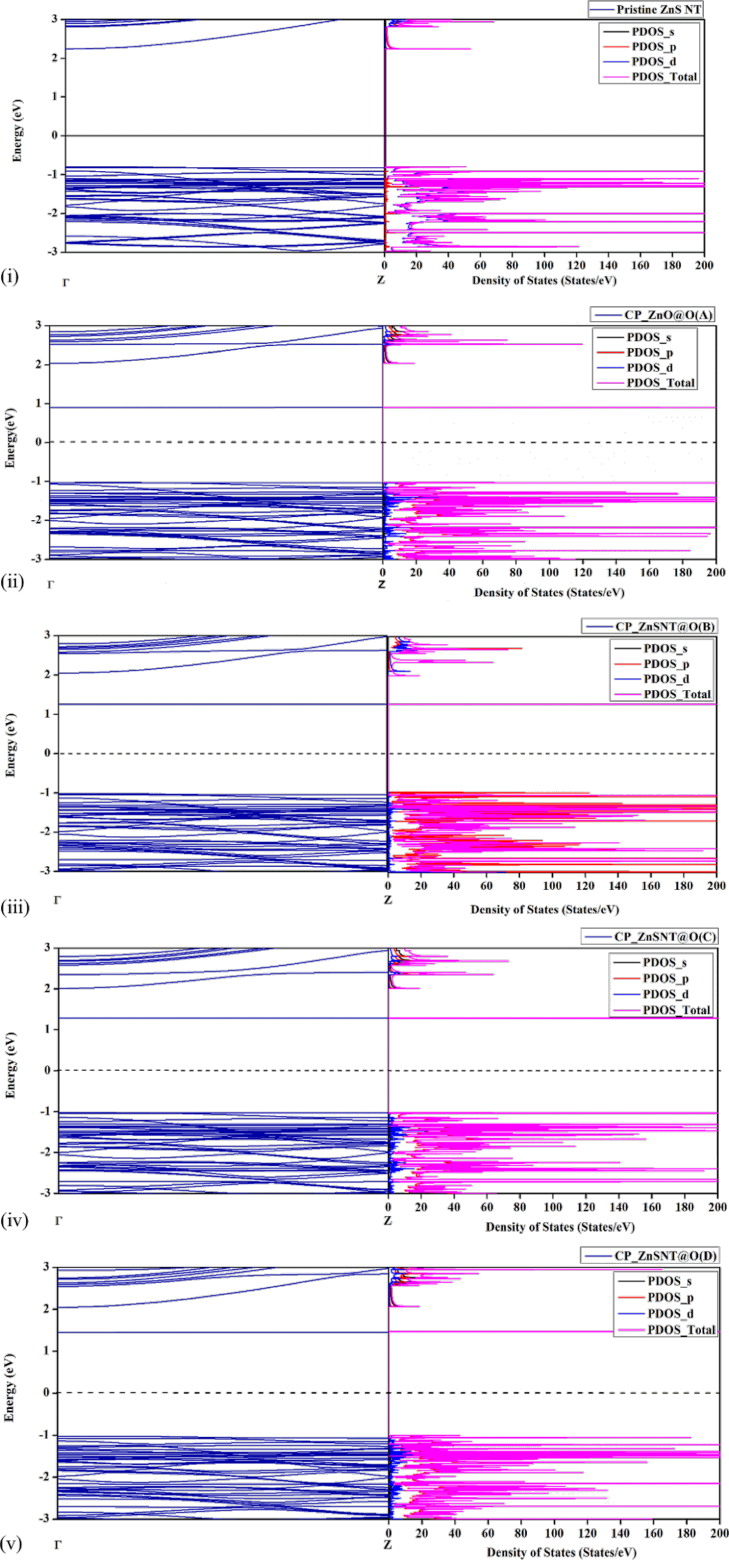
Band structure and projected density of states (PDOS) profiles of (i) pristine ZnS NT, (ii) CP adsorbed ZnS NT (Orientation A)**,** (iii) CP adsorbed ZnS NT (Orientation B), (iv) CP adsorbed ZnS NT (Orientation C), and (v) CP adsorbed ZnS NT (Orientation D).

The electronic properties of ZnS NT in the presence of CP molecules are analyzed with the help of band structure, total, and projected density of states (DOS) calculations. This analysis provides critical insights into how the adsorption of CP molecules affects the electronic behavior of the NT. The band structure and DOS profile for pristine ZnS NT and CP-adsorbed ZnS NT in orientations A, B, C, and D are shown in Figure 4.

The pristine ZnS NT exhibits a bandgap of 3.03 eV (Figure 3i), consistent with previous studies [38,40,54–58]. Upon CP adsorption, the bandgap values for orientations A, B, C, and D were reduced to 1.92, 2.27, 2.31, and 2.47 eV, respectively.

This reduction in the bandgap is evident from the band spectra and highlights the electronic interaction between CP and ZnS NT. The most prominent reduction of 40% is observed for orientation A. The decrease in the bandgap implies improved electronic conductivity of ZnS NT upon exposure to CP. The PDOS profiles show additional peaks in the ZnS NT system upon CP adsorption. Notably, the interaction of CP introduces distinct states near both the conduction and valence bands. Unlike the pristine ZnS NT, the PDOS of CP-adsorbed ZnS NTs shows new peaks around 1 eV (relative to the Fermi level), primarily consisting of states from the d orbitals of Zn and the p orbitals of S.

Based on Koopman’s theorem [59–60] the highest-occupied molecular orbital energy (HOMO) of the target molecule was employed to estimate the ionization potential (IP). In contrast, the lowest-unoccupied molecular orbital energy (LUMO) correlates with electron affinity (EA). According to the Mulliken's definition [60], the average of these two energies yields electronegativity (χ), as shown in Equation 5.


[5] $$\chi =\frac{\left(\text{EA}+\text{IP}\right)}{2}$$

Ionization potential is the energy required to remove an electron from the molecule, whereas electron affinity refers to the energy released when an electron is added to the molecule. The calculated IP and EA values for the target molecules are summarized in Table 3. Ionization potential is critical for assessing chemical reactivity; high IP values indicate chemical inertness and greater stability, whereas low IP values suggest higher molecular reactivity. The results show that the lower IP values of the target molecules make them more likely to donate electrons to the acceptor system.

**Table 3:** Ionization energy, electronegativity, and electron affinity of target CP molecules for orientations A, B, C, and D on the ZnS NT surface.

Target molecule orientation	Ionization energy (eV)	Electron affinity (eV)	Electronegativity

CP_ZnSNT@O (A)	0.611	0.905	0.758
CP_ZnSNT@O (B)	0.710	1.453	1.081
CP_ZnSNT@O (C)	0.717	1.288	1.003
CP_ZnSNT@O (D)	0.704	1.260	0.982

The percentage change in energy bandgap for CP adsorption on ZnS NT (see Table 4) is 40%, 29.6%, 27%, and 23% for orientations A, B, C, and D, respectively. Among these, the most significant variation in the energy gap is observed for orientation A. Consequently, the conductivity of ZnS NT exhibits a significant variation, demonstrating its suitability for sensing applications.

The sensitivity of the sensor is defined in terms of the change in the bandgap [61] by using the following Equation 6:


[6] $$\Delta E_{\text{g}}=\frac{\left(E_{\text{g}2}-E_{\text{g}1}\right)}{E_{\text{g}1}}*100,$$

where *E*_g1_ and *E*_g2_ are the bandgaps of the pristine ZnS NT and CP-adsorbed ZnS NT, respectively.

The recovery time [5,62–63] is influenced by the adsorption energy, as calculated by Equation 7:


[7] $$\tau =\vartheta_{0}^{-1}e^{\left(\frac{-\Delta E_{\text{ads}}}{K_{\text{B}}T}\right)},$$

where *T* is the Temperature (= 300 °C), *K*_B_ is the Boltzmann constant (8.617 × 10^−5^ eV/K), and ϑ_0_ is the attempt frequencies (= 10^12^ Hz).

The recovery time of ZnS NT for various orientations of CP adsorption is calculated and presented in Table 4. Remarkably, the sensing response and recovery time of ZnS NT towards the CP molecule for orientation A is exceptionally favorable.

**Table 4:** The adsorption energy *E*_ads_, bandgap *E*_g_, % change in bandgap Δ*E*_g_ (sensitivity), Mulliken charge transfer, and recovery time of the armchair ZnS NTs at orientations A, B, C, and D, respectively.

S.No	Orientation of molecule	Adsorption energy *E*_ads_ (eV)	Bandgap *E*_g_ (eV)	% Change in bandgap, Δ*E*_g_	Mulliken charge transfer *Q* (e)	Recovery time τ (s)

01	Pristine ZnS NT	–	3.17	–	–	–
02	CCl_3_NO_2_ - ZnS NT (A)	−0.389	1.92	40	0.073	3.533 μs
03	CCl_3_NO_2_ - ZnS NT (B)	−0.657	2.27	29.6	0.095	0.114 s
04	CCl_3_NO_2_ - ZnS NT (C)	−0.593	2.31	27	0.109	9.60 ms
05	CCl_3_NO_2_ - ZnS NT (D)	−0.440	2.47	23	0.06	25.50 μs

Notably, the recovery time of ZnS NT for orientation A is estimated as 3.5 μs at 300 K, the fastest among all previous works related to CP adsorption listed in Table 5, underscoring its exceptional potential for real-time sensing applications.

**Table 5:** Comparison of sensing response and recovery time with previous works.

Materials	Target molecules	Sensing response	Recovery time	References

Kagome phosphorene	chloropicrin, phosgene	58.28%	–	[17]
BN nanocones	chloropicrin	84.1%	37 μs at 298 K	[64]
pristine nanographenes	chloropicrin	41.1%	–	[65]
borazine-doped nanographenes	chloropicrin	39.7%	14.6 s at 350 K	[65]
silicon carbide CP@C_Si_12_C_12_ nanocluster	chloropicrin	28.8%	5.98 × 10^29^ s	[66]
CP@Si_Si_12_C_12_ Nanocluster	chloropicrin	35.4%	4.38 × 10^29^ s	[66]
green Phosphorene NT	adamsite and chloropicrin	41.4%	–	[67]
ZnS NT	chloropicrin	40%	3.5 μs at 300 K	present work

### Optical properties

**Figure 5:**
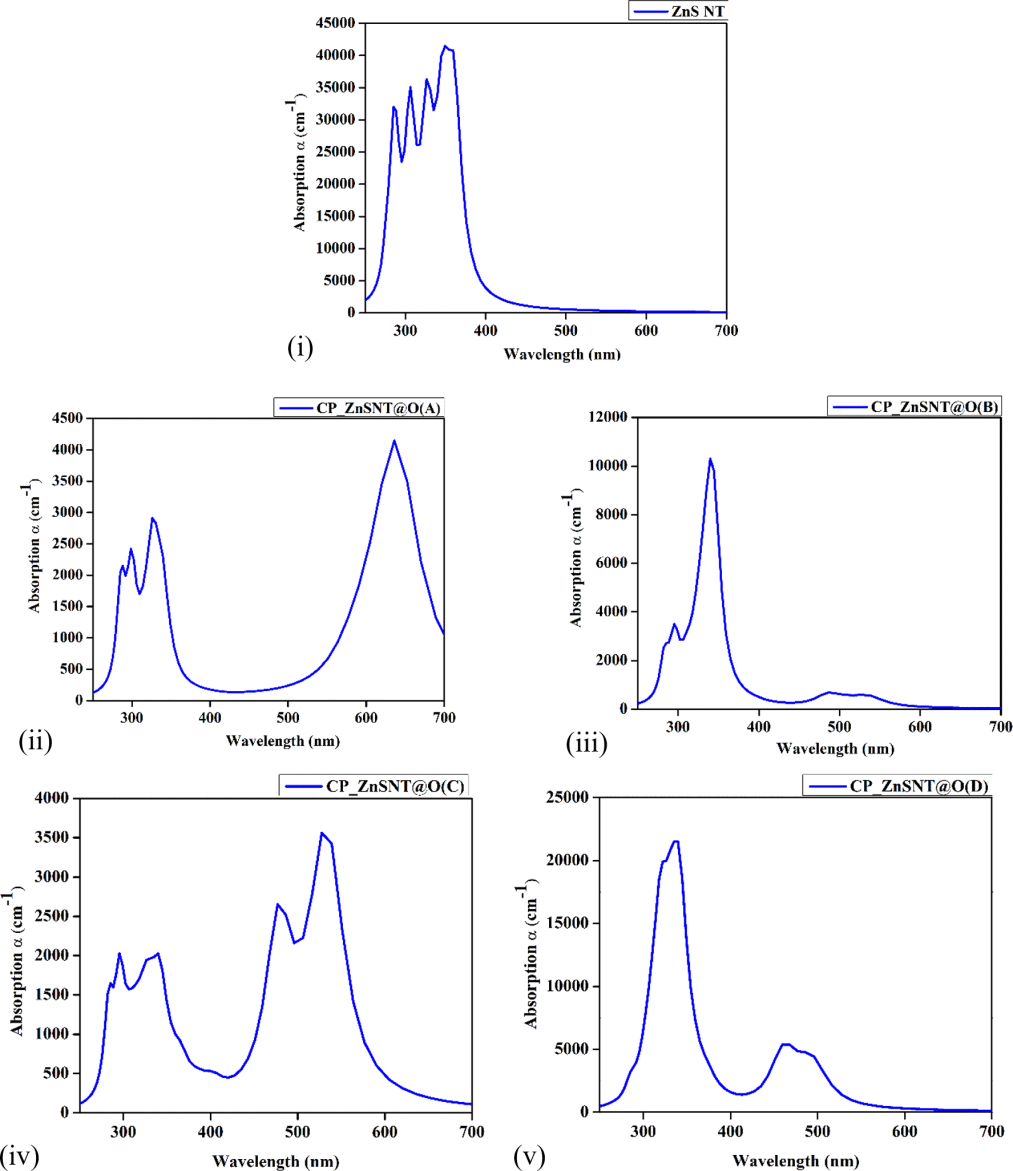
Absorption spectra (in YY-direction) for (i) pristine ZnS NT, (ii) CP adsorbed ZnS NT (Orientation A)**,** (iii) CP adsorbed ZnS NT (Orientation B), (iv) CP adsorbed ZnS NT (Orientation C), and (v) CP adsorbed ZnS NT (Orientation D).

The absorption spectra shown in Figure 5 of pristine ZnS NT and CP-adsorbed ZnS NT at orientations A, B, C, and D reveal significant variations that elucidate the interaction mechanisms between CP molecules and ZnS NTs. For pristine ZnS NTs, the spectrum displays multiple sharp peaks in the UV region (250–400 nm), with the strongest peak occurring at approximately 290–300 nm. This behavior is attributed to the intrinsic wide bandgap of ZnS (≈3.68 eV), which confines its absorption to the UV range, as confirmed by both experimental and theoretical studies on ZnS nanostructures [68]. These peaks arise from interband electronic transitions between the valence and conduction bands, reflecting the fundamental optical properties of the material. Furthermore, the absence of significant absorption in the visible region (>400 nm) underscores the lack of mid-gap states in pristine ZnS NTs. Such optical characteristics align with previous reports highlighting the suitability of ZnS nanostructures for UV photodetectors, sensors, and photocatalytic applications, where high UV sensitivity is critical [69].

Upon CP molecule adsorption, significant redshifts in the absorption spectra are observed across all orientations, indicative of bandgap narrowing caused by molecular interactions. Among the orientations, Orientation A shows the most pronounced effect, with a strong absorption peak appearing in the visible range (500–600 nm). This redshift is directly linked to the formation of mid-gap electronic states induced by charge transfer from the CP molecule to the ZnS NT. Such changes in the electronic structure are consistent with DFT studies that demonstrate adsorption-induced bandgap modulation in semiconducting nanostructures. The enhanced absorption intensity observed in Orientation A reflects strong coupling between the adsorbed CP molecule and the ZnS NT, highlighting this configuration as the most effective for modulating optical properties.

This pronounced shift into the visible range makes Orientation A particularly suitable for optical sensing applications, where small molecular interactions can induce detectable spectral changes [70]. In contrast, Orientation B exhibits a reduced absorption intensity in the UV range, with a weaker secondary peak in the visible range (400–500 nm). This suggests a weaker interaction between the CP molecule and the ZnS NT, likely due to less favorable adsorption geometry. Consequently, the bandgap modification is less pronounced than Orientation A, resulting in moderate optical tuning. Such orientation-dependent optical responses are consistent with prior studies on ZnS NTs, where adsorption sites and molecular orientation strongly influence electronic coupling and optical transitions [71]. Orientation C displays a broader absorption spectrum with multiple peaks spanning the UV (250–400 nm) and visible (400–700 nm) regions. The visible range peaks, though less intense than those in Orientation A, are broader, indicating complex interactions between the CP molecule and the ZnS NT. This could arise from the adsorption of CP molecules at multiple active sites or a combination of adsorption geometries, as previously observed in DFT-based studies on nanostructure–molecule interactions [72]. The presence of multiple optical transitions suggests moderate bandgap narrowing and indicates potential for broader spectral sensitivity, making Orientation C a versatile configuration for applications requiring multi-wavelength optical detection. Orientation D, on the other hand, shows absorption spectra similar to that of pristine ZnS NT, with only a slight redshift and weak absorption in the visible range. This suggests that the interaction between CP molecules and ZnS NT in Orientation D is minimal, resulting in negligible modifications to the electronic structure. Such weak adsorption effects are consistent with configurations where molecular interactions are dominated by van der Waals forces rather than strong charge transfer or dipole interactions [73].

The anisotropic nature of ZnS NTs plays a significant role in the observed orientation-dependent optical responses. Adsorption along orientations such as A induces stronger dipole interactions and greater charge transfer, leading to more pronounced bandgap narrowing and enhanced visible light absorption. This anisotropic behavior is consistent with theoretical and experimental studies on nanotube systems, where adsorption-induced optical changes are highly dependent on molecular orientation and the local electronic environment [74]. The redshifts observed in the visible range for CP-adsorbed ZnS NTs highlight the tunability of their optical properties, particularly in Orientation A, which exhibits the strongest response. Such behavior demonstrates the suitability of ZnS NTs as optical sensors capable of detecting CP molecules with high sensitivity and specificity.

**Figure 6:**
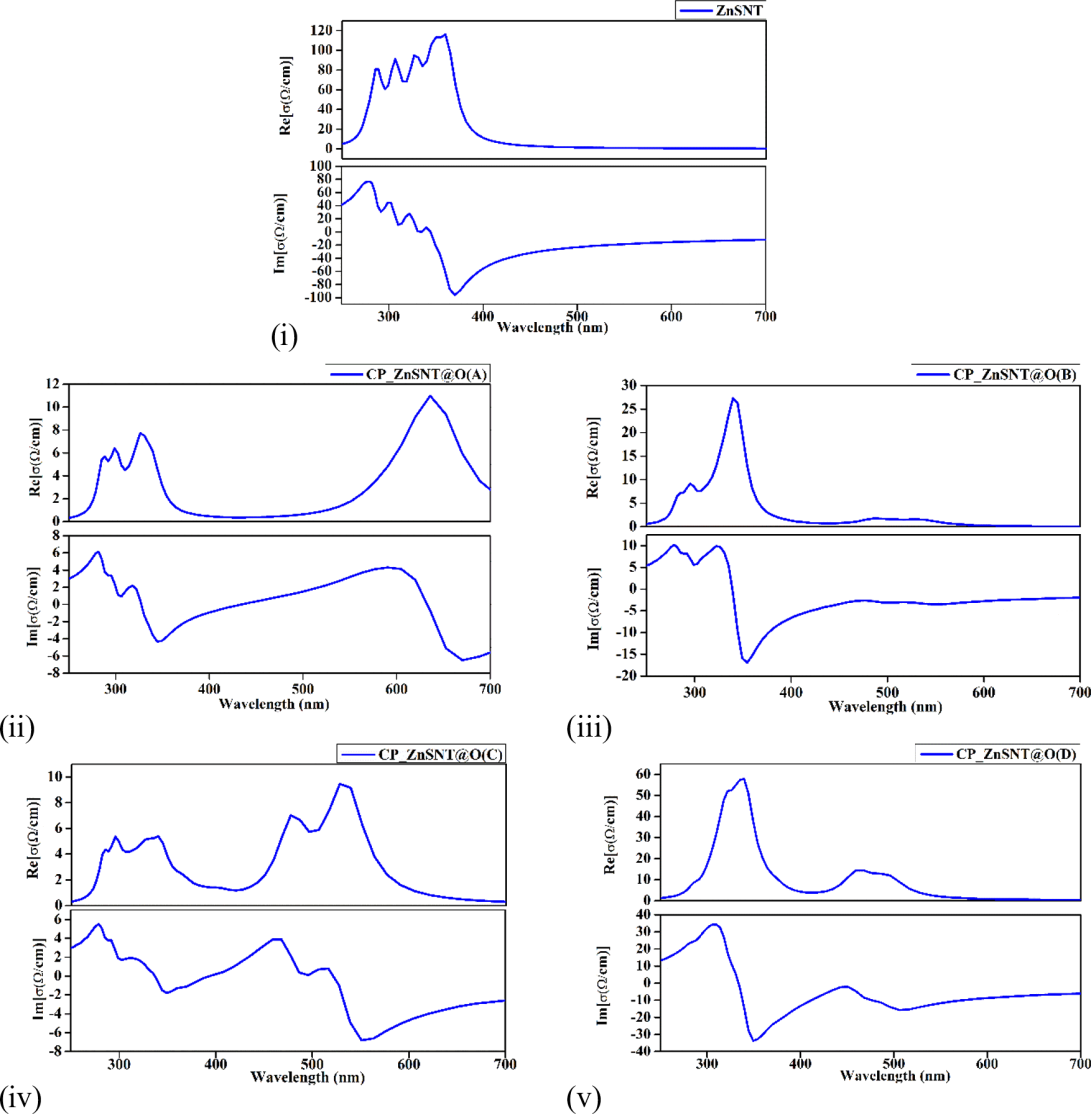
Optical conductivity (in YY-direction) for (i) pristine ZnS NT, (ii) CP adsorbed ZnS NT (Orientation A), (iii) CP adsorbed ZnS NT (Orientation B), (iv) CP adsorbed ZnS NT (Orientation C), and (v) CP adsorbed ZnS NT (Orientation D).

The optical conductivity spectra illustrated in Figure 6 of pristine ZnS NTs and CP-adsorbed ZnS N at orientations A, B, C, and D reveal significant orientation-dependent variations due to CP adsorption. Pristine ZnS NTs exhibit a dominant peak in the real part (σ_1_(ω)) at ≈395 nm, consistent with interband transitions of ZnS (≈3.68 eV), and a peak in the imaginary part (σ_2_(ω)) at ≈290 nm, representing stored energy. These UV absorption properties align with the wide bandgap of ZnS, making it suitable for UV photodetectors and photocatalysis. CP adsorption induces redshifts in σ_1_(ω) and σ_2_(ω), reflecting bandgap narrowing. Orientation A shows the most significant redshift (σ_1_(ω)) peak at ≈630 nm, attributed to mid-gap state formation due to strong charge transfer, enhancing visible light absorption for sensing applications. Orientation B exhibits a smaller redshift (≈410 nm), indicating weaker interaction. Orientation C shows multiple peaks across the ultraviolet and visible ranges, suggesting complex adsorption behavior. Orientation D exhibits negligible shifts, dominated by van der Waals forces.

These findings demonstrate the anisotropic optical behavior of ZnS NTs, with Orientation A offering the strongest potential for optical sensing. In contrast, others show moderate or limited tuning, supporting tailored optoelectronic applications.

## Conclusion

In conclusion, this work uses first-principles simulations to analyze the adsorption of chloropicrin on ZnS NT. The adsorption configurations, charge transfer, band structure, density of states, optical absorption, and optical conductivity have been systematically analyzed. The adsorption of CP induces substantial modifications in the optical and electronic properties of the NT, including a remarkable 40% reduction in the energy bandgap, a high recovery time of 3.5 μs at room temperature supported by the weak van-der-Waals-based physisorption phenomenon and significant red shift in the absorption coefficient and optical conductivity peaks. The results underscore the potential of ZnS NT as a sensor material for CP and the suitability for realizing reusable sensors.

## Data Availability

Data generated and analyzed during this study is available from the corresponding author upon reasonable request.

## AI Tools Usage

Grammarly was used only to improve the English language used in the article.

## References

[1] Pesonen M, Vähäkangas K (2020). Toxicol Lett.

[2] Zhou Y, Ye Z-X, Huang H, Liu Y D, Zhong R (2021). J Hazard Mater.

[3] Sparks S E, Quistad G B, Casida J E (1997). Chem Res Toxicol.

[4] Mukherjee S, Suntres Z, Stone W, Smith M, Das S, Ward P, Guo R-F, Romano J A, Lukey B J, Salem H (2007). Vesicants and Oxidative Stress. Chemical Warfare Agents.

[5] Muir B, Carrick W A, Cooper D B (2002). Analyst.

[6] Gaskin S, Heath L, Pisaniello D, Edwards J W, Logan M, Baxter C (2017). Toxicol Ind Health.

[7] Goldman L R, Mengle D, Epstein D M, Fredson D, Kelly K, Jackson R J (1987). West J Med.

[8] Kumar A, Kumar M, Bhatt V, Mukherjee S, Kumar S, Sharma H, Yadav M K, Tomar S, Yun J-H, Choubey R K (2021). Sens Actuators, A.

[9] Zhu L-Y, Ou L-X, Mao L-W, Wu X-Y, Liu Y-P, Lu H-L (2023). Nano-Micro Lett.

[10] Aparna S M, Rakhi R B (2025). Mater Sci Eng, B.

[11] Fan Y, Song L, Wang W, Fan H (2025). Nanomaterials.

[12] Tavakoli Z, Sheikhi M, Shahab S, Kaviani S, Sheikhi B, Kumar R (2022). Main Group Chem.

[13] Kaviani S, Khajavian M, Piyanzina I, Nedopekin O V, Tayurskii D A (2024). J Mol Graphics Mod.

[14] Kaviani S, Tayurskii D A, Nedopekin O V, Piyanzina I (2022). J Phys Chem Solids.

[15] Fang X, Zhai T, Gautam U K, Li L, Wu L, Bando Y, Golberg D (2011). Prog Mater Sci.

[16] Feng W, Yuan J, Zhang L, Hu W, Wu Z, Wang X, Huang X, Liu P, Zhang S (2020). Appl Catal, B.

[17] Princy Maria J, Bhuvaneswari R, Nagarajan V, Chandiramouli R (2021). Chem Phys Lett.

[18] Tsai Y-S, Lin X, Wu Y S, Chen H, Han J (2021). IEEE Sens J.

[19] Jogender, Badhani B, Mandeep, Kakkar R (2020). J Mol Graphics Mod.

[20] Hazrati M K, Bagheri Z, Bodaghi A (2017). Phys E (Amsterdam, Neth).

[21] Cai X, Deng S, Li L, Hao L (2020). J Comput Electron.

[22] Soliman K A, Aal S A (2022). Mater Sci Semicond Process.

[23] Sharma A, Khan M S, Srivastava A, Khan M S, Husain M (2019). AIP Conf Proc.

[24] Noei M (2017). Vacuum.

[25] Saedi L, Jameh-Bozorghi S, Maskanati M, Soleymanabadi H (2018). Inorg Chem Commun.

[26] Soleymanabadi H, Kakemam J (2013). Phys E (Amsterdam, Neth).

[27] Rastegar S F, Hadipour N L, Tabar M B, Soleymanabadi H (2013). J Mol Model.

[28] Jameh-Bozorghi S, Soleymanabadi H (2017). Phys Lett A.

[29] Rostami Z, Pashangpour M, Moradi R (2017). J Mol Graphics Mod.

[30] Nayebzadeh M, Peyghan A A, Soleymanabadi H (2014). Phys E (Amsterdam, Neth).

[31] Shabavi Z M, Shakerzadeh E, Yadav T, Tahmasebi E, Kaviani S, Anota E C (2024). Comput Theor Chem.

[32] Vishwkarma A K, Yadav T, Shakerzadeh E, Goswami S, Garai S, Vetrivelan V, Adam J, Pathak A (2025). J Mol Graphics Mod.

[33] Yadav T, Vishwkarma A K, Shakerzadeh E, Adam J, Kumar P, Vaithiyanathan V, Pathak A, Nguyen M T (2025). Colloids Surf, A.

[34] Shabavi Z M, Shakerzadeh E, Yadav T, Anota E C (2024). Diamond Relat Mater.

[35] Yadav T, Shakerzadeh E, Vishwkarma A K, Singh P K, Pathak A, Chakroborty S, Pandey F P, Moharana S, Kumar R (2023). Diamond Relat Mater.

[36] Pradyumn, Barman P B, Sil A, Hazra S K (2025). J Alloys Compd.

[37] D’Amico P, Calzolari A, Ruini A, Catellani A (2017). Sci Rep.

[38] Farhangfar S, Yang R B, Pelletier M, Nielsch K (2009). Nanotechnology.

[39] Kumar M, Lamba V K (2019). J Nanosci, NanoEng Appl.

[40] Khan M S, Srivastava A, Chaurasiya R, Khan M S, Dua P (2015). Chem Phys Lett.

[41] Zhang H, Zhang S, Pan S, Li G, Hou J (2004). Nanotechnology.

[42] Zhang X, Zhao M, He T, Li W, Lin X, Wang Z, Xi Z, Liu X, Xia Y (2008). Solid State Commun.

[43] Park S, Sun G-J, Kheel H, Ko T, Kim H W, Lee C (2016). Appl Phys A: Mater Sci Process.

[44] Zhang X, Zhao M, Yan S, He T, Li W, Lin X, Xi Z, Wang Z, Liu X, Xia Y (2008). Nanotechnology.

[45] Zhu Y-F, Zhang J, Xu L, Guo Y, Wang X-P, Du R-G, Lin C-J (2013). Phys Chem Chem Phys.

[46] Zhang W, Wang S, Wang Y, Zhu Z, Gao X, Yang J, Zhang H x (2015). RSC Adv.

[47] Zhang W, Feng C, Yang Z (2012). Sens Actuators, B.

[48] Giri A K, Charan C, Ghosh S C, Shahi V K, Panda A B (2016). Sens Actuators, B.

[49] Smidstrup S, Markussen T, Vancraeyveld P, Wellendorff J, Schneider J, Gunst T, Verstichel B, Stradi D, Khomyakov P A, Vej-Hansen U G (2020). J Phys: Condens Matter.

[50] Ernzerhof M, Perdew J P (1998). J Chem Phys.

[51] Vig A, Doan E, Yang K (2023). Nanomaterials.

[52] Bafekry A, Naseri M, Faraji M, Fadlallah M M, Hoat D M, Jappor H R, Ghergherehchi M, Gogova D, Afarideh H (2022). Sci Rep.

[53] Malyi O I, Sopiha K, Kulish V V, Tan T L, Manzhos S, Persson C (2015). Appl Surf Sci.

[54] Zhai T, Gu Z, Ma Y, Yang W, Zhao L, Yao J (2006). Mater Chem Phys.

[55] Pal S, Goswami B, Sarkar P (2007). Indian J Phys.

[56] Krainara N, Limtrakul J, Illas F, Bromley S T (2011). Phys Rev B: Condens Matter Mater Phys.

[57] Chen H, Liu C (2013). Chin J Comput Phys.

[58] An Q, Meng X, Xiong K, Qiu Y (2017). Sci Rep.

[59] Tsuneda T, Song J-W, Suzuki S, Hirao K (2010). J Chem Phys.

[60] Banibairami T, Jamehbozorgi S, Ghiasi R, Rezvani M (2020). Russ J Phys Chem A.

[61] Hadipour N L, Ahmadi Peyghan A, Soleymanabadi H (2015). J Phys Chem C.

[62] Rostami Z, Soleymanabadi H (2017). J Mol Liq.

[63] Chen X, Wang T, Han Y, Lv W, Li B, Su C, Zeng M, Yang J, Hu N, Su Y (2021). Sens Actuators, B.

[64] Vessally E, Moladoust R, Mousavi-Khoshdel S M, Esrafili M D, Hosseinian A, Edjlali L (2018). Struct Chem.

[65] Hosseinian A, Vessally E, Babazadeh M, Edjlali L, Es’haghi M (2018). J Phys Chem Solids.

[66] Agwamba E C, Chukwuemeka K, Louis H, Okon G A, Eni D I, Manicum A-L E (2024). Silicon.

[67] Nagarajan V, Chandiramouli R (2020). Chem Phys.

[68] Yousfi A, El farri H, Ait Labyad N, Benaicha I, Mhalla J, El-Habib A, Laghchim E, Raidou A, Bendoumou A, Nouneh K (2025). Opt Quantum Electron.

[69] Xu H, Fan Z, Liu Q, Li L (2023). Open Chem.

[70] Michos F I, Sigalas M M (2018). J Appl Phys.

[71] Sakr M A S, Saad M A, Abdelsalam H, Abd-Elkader O H, Aleya L, Zhang Q (2023). J Electron Mater.

[72] Tarish S, Al-Haddad A, Xu R, Cao D, Wang Z, Qu S, Nabi G, Lei Y (2016). J Mater Chem C.

[73] Es-Smairi A, Fazouan N, Bziz I, El Houssine A (2018). DFT Study of Structural, Electronic and Optical Properties of ZnS Phases. 2018 6th International Renewable and Sustainable Energy Conference (IRSEC).

[74] Dengo N, Vittadini A, Natile M M, Gross S (2020). J Phys Chem C.

